# The Association Between Electronic Cigarette Use During Pregnancy and Unfavorable Birth Outcomes

**DOI:** 10.7759/cureus.26748

**Published:** 2022-07-11

**Authors:** Alexandra Galbo, Nicole Izhakoff, Connor Courington, Grettel Castro, Juan Lozano, Juan Ruiz-Pelaez

**Affiliations:** 1 Department of Translational Medicine, Florida International University, Herbert Wertheim College of Medicine, Miami, USA; 2 Department of Medical and Population Health Sciences Research, Florida International University, Herbert Wertheim College of Medicine, Miami, USA; 3 Kangaroo Mother Care Senior Researcher, Kangaroo Foundation, Bogota, COL

**Keywords:** prams, low birth weight, preterm birth, pregnancy, electronic cigarettes, e-cigs

## Abstract

Introduction and objective

While the use of electronic cigarettes (e-cigarettes) continues to gain popularity amongst consumers, literature focusing on the safety and risks of e-cigarette usage remains scarce. Literature focused on the potential effects of e-cigarette use on fetal development is particularly limited. The objective of this study is to investigate the association between the use of e-cigarettes during pregnancy and unfavorable birth outcomes.

Methods

A retrospective cohort using secondary data analysis was conducted from the Pregnancy Risk Assessment Monitoring System (PRAMS) 2016-2017 Phase 8 survey. This database contains both state-specific and population-based information on maternal attitudes and experiences before, during, and shortly after pregnancy. Female participants in the study were initially found through each state’s birth certificate file. Eligible women included those who have had a recent live birth. Data collection procedures and instruments were standardized to allow comparisons between states. The independent variable was self-reported use of any e-cigarette products during pregnancy. The dependent variable was dichotomized into the presence of at least one unfavorable birth outcome (preterm birth, low birth weight, or extended postnatal hospital stay for the newborn) or the absence of all. Binary logistic regression analysis was used to calculate adjusted odds ratios (aOR) and corresponding 95% confidence intervals (CI).

Results

A total of 71,940 women were included in our study. After adjusting for age, race, ethnicity, insurance, maternal education, prenatal care, physical abuse during pregnancy, and complications during pregnancy, the odds of unfavorable birth outcomes increase by 62% among women who reported e-cigarette use during pregnancy versus women who did not (aOR 1.62, 95%CI 1.16-2.26, p-value 0.005).

Conclusions/implications

Moving forward, it is imperative for consumers to understand the implications of using e-cigarettes, such as the increased risk of unfavorable birth outcomes associated with use during pregnancy. Moreover, healthcare providers, particularly obstetricians, should be encouraged to communicate this novel information to at-risk patients. Overall, researchers must continue to study the long-term effects of e-cigarettes, including those on fetal development, as there is still much to be uncovered.

## Introduction

Proper fetal growth is fundamental to an individual’s long-term development. Disruption of developmental pathways may cause significant, unfavorable birth outcomes including low birth weight, preterm birth, and an extended postnatal stay for newborns. 

Fetal growth is primarily influenced by the fetal environment and the adequate delivery of maternal nutrients in utero [1]. Interfering with these processes can lead to fetal growth restriction and, ultimately, a low birth weight. Causes of low birth weight in humans are multifactorial and include adolescent pregnancy, maternal weight below 50 kg, family dysfunction, malnutrition (especially during pregnancy), and others. Infants suffering from low birth weight have up to twelve times higher perinatal mortality, three times higher morbidity, and an increased risk of disease later in life when compared to those with the appropriate birth weight for gestational age [1].

Preterm birth is defined as childbirth occurring prior to 37 completed weeks or 259 days of gestation [2]. While the etiology of preterm birth is often considered to be multifactorial, several causal factors have been identified, such as medical conditions of the mother or fetus, genetic influences, environmental exposure, infertility treatments, behavioral and socioeconomic factors, and iatrogenic prematurity [2]. Premature birth rates in developed countries have notably risen over the past several decades, with 5-7% of live births occurring prior to full gestation [2]. Fetal prematurity can translate to long-term sequelae, such as an increased risk of learning disabilities, respiratory illnesses, sensory deficits, and cerebral palsy when compared to children of normal gestational age. Preterm birth is linked to increased morbidity in adulthood, ultimately leading to increased physical, psychological and economic costs [2].

The amount of time that a newborn spends in the hospital after birth may be used as an indicator of its health. Healthy term newborns may be ready for discharge just 48 hours after birth, if delivered vaginally, or 96 hours after birth if delivered by cesarean section [3]. Problems related to the transition from intrauterine to extrauterine environments may become apparent during the first 12 hours of birth [3]. Furthermore, detection of jaundice, cardiac lesions, gastrointestinal obstructions, and other problems may require even longer periods of observation. In serious cases of neonatal complications, the newborn may be sent to the Neonatal Intensive Care Unit for extensive therapy, leading to longer hospital stays.

Current research suggests the use of traditional cigarettes during pregnancy has been associated with unfavorable birth outcomes, including pre-term delivery, low birth weight, miscarriage, and impaired fetal growth; such outcomes may impact long-term health [4]. Many of these unfavorable birth outcomes are thought to be related to fetal exposure to nicotine during pregnancy. Nicotine is an active constituent in traditional cigarettes and induces their addictive potential. Nicotine is also present in electronic cigarettes.

Electronic cigarettes (e-cigarettes) are battery-powered, hand-held vaporizing devices that simulate the act of cigarette smoking by providing the user with a controlled dose of vaporized nicotine [5]. Since the introduction of e-cigarettes to the U.S. market in 2007, the U.S. has become the largest and fastest-growing region for e-cigarettes in the world, with an estimated industry value of approximately $10 billion [4]. E-cigarette sales and reported usage have been increasing annually within the United States.

The objective of this study was to investigate the association between women who used e-cigarettes during pregnancy and unfavorable birth outcomes (preterm birth, low birth weight, extended postnatal hospital stay for the newborn) in comparison to women who did not use e-cigarettes during pregnancy. We hypothesize that there is a significant association between women who use e-cigarettes during pregnancy and unfavorable birth outcomes, as compared to non-smoking pregnant women.

## Materials and methods

**Figure 1:**
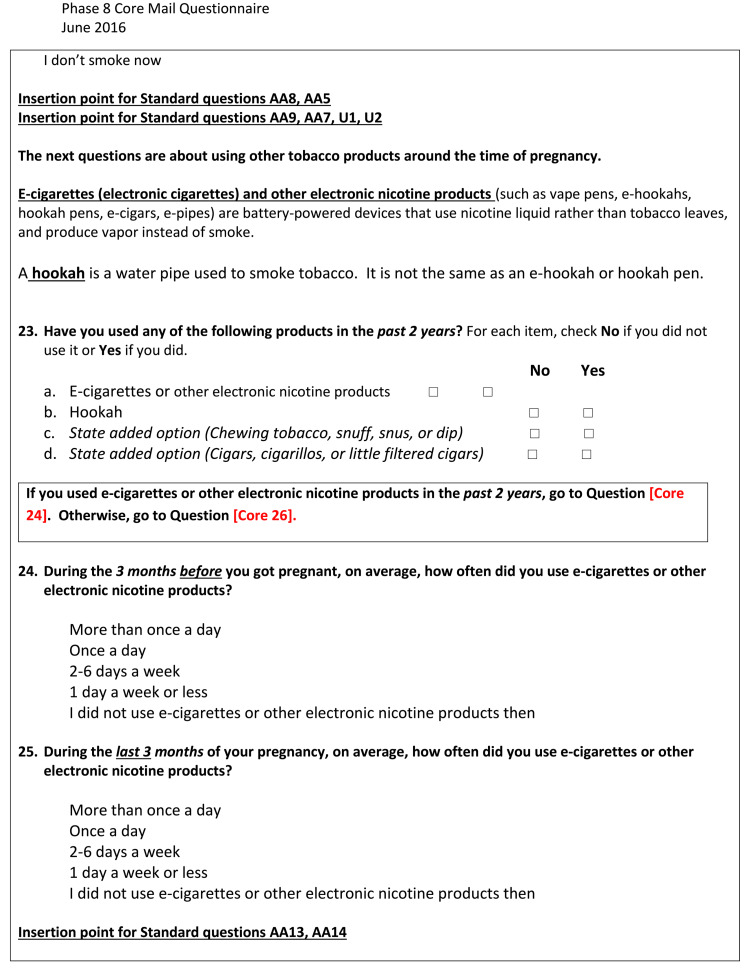
PRAMS Phase 8 Core Questionnaire, Q. 25 Source: United States Centers for Disease Control and Prevention (CDC) [6].

A retrospective cohort study was conducted based on secondary analysis using data from the Pregnancy Risk Assessment Monitoring System (PRAMS) Phase 8 core survey given between 2016-2017. Forty-seven of the 50 U.S. states, Puerto Rico, and the District of Columbia participated in this phase of the survey, which has been provided in the Appendices (Figure 1) for reference [6]. The PRAMS database contains both state-specific and population-based information on maternal attitudes and experiences before, during, and after pregnancy. Data are collected through a two-part questionnaire consisting of core questions asked by all states participating in the project as well as U.S. Centers for Disease Control and Prevention (CDC)-developed, supplemental questions. Women participating in the study are found through each state’s birth certificate file. Data collection procedures and instruments are standardized to allow comparisons between states. Specific responses necessary for our study include the participant’s frequency of e-cigarette use during pregnancy, the infant’s gestational age and weight at the time of birth, and the length of the infant’s hospital stay postnatal.

Study population and design

The sample of our study included women in the United States who completed the Phase 8 Core Questions from the PRAMS survey in 2016 and 2017 [6]. This data was requested from the CDC for research utilization. As this study is a retrospective cohort analysis and qualifies as secondary research, it was exempt from IRB review. The inclusion criteria consisted of all women who completed the question regarding e-cigarette use in the last three (3) months of their pregnancy. Participants were excluded from the study if they failed to complete the questions regarding the use of e-cigarettes during pregnancy or the outcomes of interest (preterm birth, low birth weight, or length of postnatal hospital stay for the newborn). As the PRAMS questionnaire does not ask about the infant’s gestational age or weight at birth, we collected this information through the birth certificate information available in the PRAMS Analytic Research File. Any women or birth certificates that did not have information regarding these outcomes were excluded from the study.

The independent variable of this study was the use of e-cigarettes during pregnancy as detailed in Q25 of the Phase 8 Core PRAMS survey, “During the last 3 months of your pregnancy, on average, how often did you use e-cigarettes or other electronic nicotine products?” The dependent variable of interest was unfavorable birth outcomes: preterm birth, low birth weight, and an extended postnatal hospital stay for the newborn. Preterm birth was based on the World Health Organization (WHO) criteria of an infant born at less than 37 completed weeks of gestation [7]. Extended postnatal hospital stay for the newborn was based on the American Academy of Pediatrics criteria, defined as a period greater than 48 hours for vaginal deliveries and greater than 96 hours for deliveries via cesarean section [3]. Low birth weight was based on the WHO criteria of an infant weighing less than 2500 g at birth [7].

There were several potential confounders and effect modifiers for this study, including age, race, Hispanic ethnicity, insurance, maternal education, Kotelchuck index, physical abuse during pregnancy, and complications during pregnancy including gestational diabetes, high blood pressure, and clinical depression. Of note, maternal education was delineated as those who 1) in/did not finish high school, 2) finished high school, or 3) higher education. This organization is intended to objectively categorize data based on education demographics, irrespective of the participant's age. Other variables considered confounders included traditional tobacco, drug or alcohol use and congenital anomalies, pregnancy with multiple gestation, education level, inadequate nutrition, and medication usage. These confounders could not be measured as they were not addressed in the core questionnaire.

Statistical analyses

The sample was assessed for baseline characteristics. Nominal variables were summarized using percentages while continuous variables were analyzed using means and standard deviations (in normal distribution), or medians and interquartile ranges (in distributions that were not normal). A descriptive analysis of each variable was performed prior to utilizing a bivariate analysis to assess the association between potential confounders and the exposure, and if there was an association between the independent variable or potential confounders and the outcome. This portion of the analysis utilized t-tests and χ2 tests, as needed. Odds ratios and confidence intervals were calculated for each variable based on the bivariate analysis. A co-linearity analysis was then performed to ensure no association between covariates that could serve as confounders. A multivariate logistic regression analysis was performed to control for any statistically significant confounders. The odds ratios were then adjusted based on the strength of the potential confounders retained for the multivariate model. The values of these adjusted odds ratios were then compared to the values of the odds ratios that were calculated using the bivariate analysis. Additionally, our analysis included weighted percentages to account for the adequate representation of certain groups.

## Results

Of the 73,162 postpartum women who participated in the PRAMS survey, 1,222 did not respond to the questions regarding e-cigarette use during pregnancy (Q25) or newborn’s length of hospital stay (Q31), and were thus excluded. The final sample size consisted of 71,940 postpartum women in the United States who participated in the PRAMS survey in 2016.

Out of the 71,940 postpartum women included in our study, 859 (1.2%) self-reported e-cigarette use during pregnancy. Baseline characteristics between pregnant e-cigarette users vs non-users are depicted in Table 1. E-cigarette users during pregnancy were more often young (36.2% under 25 years old vs 23.3% in non-users), White (85.9% vs 69.4%), and Non-Hispanic (93% vs 81.2%). Typical users were found to have Medicaid as their primary insurance provider (71.8% vs 40.6%), had not completed higher education (38.6 vs 63.4%), had not received adequate prenatal care (20.2% vs 12.4%), were victims of physical abuse during pregnancy (19.4% vs 5.1%), and reported a clinical diagnosis of depression (38.1% vs 11.5%).

**Table 1:** Baseline Characteristics of Post-Partum Women Who Used E-Cigarettes During Pregnancy in PRAMS Database N = sample size; PRAMS: Pregnancy Risk Assessment Monitoring System

Characteristics	E-cigarette use during pregnancy				p-value
	Yes = 859		No = 71,081		
	N	%	N	%	
Age (years)					<0.001
<19	81	10.4	3,544	4.7	
20-24	220	25.8	13,369	18.6	
25-34	465	53.0	41,418	58.9	
≥35	92	10.8	12,749	17.8	
Race					<0.001
White	610	85.9	40,621	69.4	
African American	85	5.9	13,028	14.9	
Asian	8	0.6	4,828	6.0	
Other	116	7.6	10,254	9.6	
Ethnicity Hispanic	66	7.0	12,911	19.8	<0.001
Insurance					<0.001
Medicaid	627	71.8	31,017	40.6	
Private	174	24.4	34,957	53.0	
Self-pay	10	0.9	1,754	2.7	
Other	35	2.9	2,831	3.6	
Maternal Education					<0.001
In/did not finish high school	165	20.3	9,198	12.5	
Finished high school	350	41.1	17,001	24.1	
Higher education	335	38.6	44,176	63.4	
Kotelchuck Index					<0.001
Adequate plus	274	28.1	24,553	31.7	
Adequate	291	42.6	28,722	45.3	
Intermediate	88	9.0	7,104	10.7	
Inadequate	180	20.2	8,305	12.4	
Abuse during pregnancy	87	19.4	2,175	5.1	<0.001
Complications during pregnancy					
Gestational diabetes	56	5.5	6,967	9.0	0.023
High blood pressure	131	13.8	10,513	12.0	0.302
Depression	332	38.1	9,148	11.5	<0.001

When assessing for unadjusted and adjusted associations between e-cigarette use during pregnancy and unfavorable birth outcomes in postpartum women, several significant results were identified (Table 2). Notably, after adjusting for confounders, the odds of unfavorable birth outcomes increased by 62% among women who reported e-cigarette use during pregnancy versus women who did not (adjusted Odds Ratio (aOR): 1.62, 95%CI:1.16-2.26, p-value=0.005). Furthermore, after adjustment, characteristics associated with significant odds of unfavorable birth outcomes included ages <19 years old, being African-American or Asian, those receiving adequate plus prenatal care, and those who reported having high blood pressure during pregnancy (Table 2). Contrastingly, the odds of unfavorable birth outcomes decreased by 12% among women who received some level of higher education versus women who completed a high school education (aOR: 0.88, 95%CI:0.79-0.98, p-value=0.022).

**Table 2:** Unadjusted and Adjusted Associations Between E-Cigarette Use During Pregnancy and Unfavorable Birth Outcomes in Post-Partum Women OR = odds ratio

Characteristics	Unadjusted		Adjusted	
	OR (95% CI)	p-value	OR (95% CI)	p-value
E-cigarette use during pregnancy				
Yes	1.67 (1.35, 2.10)	<0.001	1.62 (1.16, 2.26)	0.005
No	reference	--	reference	--
Age (years)				
≤19	1.96 (1.74, 2.21)	<0.001	1.6 (1.31, 1.97)	<0.001
20-24	1.37 (1.29, 1.47)	<0.001	1.19 (1.06, 1.33)	0.002
25-34	reference	--	reference	--
≥35	0.97 (0.91, 1.04)	0.37	0.91 (0.82, 1.02)	0.111
Race				
White	reference	--	reference	--
African American	1.77 (1.65, 1.90)	<0.001	1.61 (1.43, 1.81)	<0.001
Asian	1.17 (1.06, 1.30)	0.002	1.38 (1.15, 1.66)	0.001
Other	1.43 (1.31, 1.55)	<0.001	1.18 (1.01, 1.38)	0.035
Ethnicity Hispanic	1.23 (1.16, 1.32)	<0.001	1.1 (0.98, 1.24)	0.11
Insurance				
Medicaid	1.65 (1.56, 1.74)	<0.001	1.22 (1.11, 1.35)	<0.001
Private	reference	--	reference	--
Self-pay	1.52 (1.28, 1.82)	<0.001	1.38 (1.02, 1.87)	0.037
Other	1.46 (1.26, 1.70)	<0.001	1.47 (1.15, 1.87)	0.002
Maternal Education				
In/did not finish high school	1.11 (1.02, 1.21)	0.021	0.96 (0.83, 1.12)	0.595
Finished high school	reference	--	reference	--
Higher education	0.66 (0.62, 0.71)	<0.001	0.88 (0.79, 0.98)	0.022
Kotelchuck Index				
Adequate plus	2.08 (1.96, 2.21)	<0.001	1.93 (1.76, 2.12)	<0.001
Adequate	reference	--	reference	--
Intermediate	1.2 (1.09, 1.32)	<0.001	1.09 (0.94, 1.27)	0.252
Inadequate	1.84 (1.69, 2.01)	<0.001	1.39 (1.21, 1.60)	<0.001
Abuse during pregnancy	1.41 (1.22, 1.64)	<0.001	1.12 (0.95, 1.32)	0.181
Complications during pregnancy				
Gestational diabetes	1.22 (1.12, 1.33)	<0.001	1.06 (0.92, 1.22)	0.402
High blood pressure	2.09 (1.95, 2.24)	<0.001	1.92 (1.72, 2.16)	<0.001
Depression	1.45 (1.35, 1.56)	<0.001	1.09 (0.97, 1.23)	0.15

## Discussion

Research establishes that e-cigarettes are perceived by many as being both a safer alternative to traditional tobacco cigarettes and safe for use during pregnancy [4]. With the rise of e-cigarettes, a significant need exists for more research regarding their potential side effects. Current research from animal models indicates an association between nicotine consumption in e-cigarette use and impaired fetal development. However, due to a lack of literature on this topic involving human subjects, the true causal effect remains unknown [8]. There have been several literature reviews examining the relationship between pregnant women and their perceptions and usage of e-cigarettes. The data regarding the association between the use of e-cigarettes during pregnancy and unfavorable birth outcomes is also growing. According to Cardenas et. al, the use of electronic nicotine delivery systems is associated with an increased fetal risk of small-for-gestational-age birth [9]. Contrastingly, other researchers have found results indicating the birthweight of infants born to electronic cigarette users is similar to that of non-smokers [10]. This gap in knowledge, although shrinking, may be causing physicians to provide patients with inaccurate information. Thus, there is an increasing need for education regarding e-cigarettes among physicians and patients. We hope that our findings can help ascertain the potential fetal effects related to e-cigarette use as well as guide future patient education regarding prenatal development and reproductive health. There currently is a lack of literature focusing on e-cigarette use specific to its implications on health outcomes. This paucity of information poses a greater risk in vulnerable populations, such as pregnant women. After controlling for various confounding variables, our study found that women using e-cigarettes during pregnancy have a 62% increased risk of unfavorable birth outcomes, compared to women who do not use e-cigarettes. 

There are conflicting results among studies assessing the association between the use of e-cigarettes during pregnancy and unfavorable birth outcomes, specifically, low birth weight. Note, however, that the studies determining these results were limited by sample size. While research conducted by Cardenas et. al and McDonnell et. al included 232 and 413 women, respectively, our study possesses a greater degree of appreciable validity due to its remarkably larger sample size [9,10].

Due to the variety of baseline characteristics included in the study, numerous secondary outcomes became apparent during data analysis. Secondary outcomes noted include significant associations between unfavorable birth outcomes and women with high blood pressure during pregnancy (aOR=1.92, adjusted p-value<0.0001) and women ≤19 years old (aOR=1.60, adjusted p-value<0.0001). Additionally, we determined that women receiving adequate plus prenatal care (as defined by the Kotelchuck Index) were more prone to unfavorable birth outcomes (aOR=1.93, adjusted p-value<0.0001). The Kotelchuck Index classifies prenatal care based on the number of pre-birth office visits. Thus, we believe this association is likely because those who are receiving adequate plus prenatal care may have additional comorbidities or fetal risk factors requiring an increased number of office visits to ensure an appropriate level of care. Further, within our study population, the odds of unfavorable birth outcomes decrease by 9% among women 35+ years old compared with women 25-34 years old. However, studies suggest that women 35+ years old are of advanced maternal age and are therefore at an increased risk of having adverse perinatal outcomes including preterm delivery and low birth weight babies [11]. Upon further analysis, we found the association between unfavorable birth outcomes and women 35+ years old to be insignificant (adjusted p-value=0.111). 

Our study used the PRAMS Phase 8 Database representing approximately 83% of all U.S. live births in 2016 and 2017. Using this database, we had access to a large sample for our analysis and were not required to follow up with any study participants. The survey included questions that addressed potential confounders to our research hypothesis. We subsequently were able to control for these confounders during analysis. Following our exclusion criteria, participants who did not respond to questions related to our covariates, exposure, or outcome, were not included to minimize bias; resulting in 71,940 participants. Additionally, as our analysis included weighted percentages to account for the adequate representation of certain groups, our study sample should be generalizable to the majority of U.S. postpartum women.

While consumers widely view e-cigarette use during pregnancy as a safer alternative to tobacco or nicotine use, our research suggests that e-cigarette use during pregnancy is associated with unfavorable birth outcomes [4]. Such outcomes bear the heaviest significance in the fields of obstetrics, gynecology, and pediatric medicine, prompting an increased need for screening.

To maximize the efficacy of care, physicians are responsible for providing comprehensive counseling to their patients. For women who are pregnant or likely to become pregnant, appropriate comprehensive health screenings and evidence-based recommendations are paramount in establishing early and adequate prenatal care. Unfortunately, according to a literature review conducted by Whittington et. al, one study details that out of 475 surveyed obstetrician-gynecologists (OBGYN), less than 53% (n=252) reported consistently screening patients for exposure to noncombustible tobacco products, such as e-cigarettes. Moreover, while assessing a similar study, 13.5% of OBGYNs reported that they consider e-cigarettes to have no adverse health effects and 66% said that they would be receptive to learning more about the health effects of e-cigarettes [12]. The effects of noncombustible tobacco products is a topical matter ripe for further research and analysis. 

Physicians may apply the results of our study via the implementation of enhanced screening for pregnant women with a social history of current or past e-cigarette use. Special attention should also be paid to those fitting the most frequent characteristics of e-cigarette users. Increasing awareness of the risks of e-cigarette usage during pregnancy within the medical community will ultimately promote increased action and primary prevention. Finally, pediatricians may be able to apply our results in everyday practice, as pediatric patients are prone to the long-term use of and potential addiction to e-cigarettes. 

This study presented predictable limitations. The question used to identify participants using e-cigarettes during pregnancy, specifically, asks about use during “the last 3 months of pregnancy.” Therefore, we do not have specifics on the amount or duration of e-cigarette use throughout the entirety of the pregnancy. Additionally, we did not have information regarding alcohol or other drug use by participants during pregnancy, as this information was obtained supplementally. It is widely accepted that maternal alcohol, traditional tobacco, and drug use are associated with unfavorable birth outcomes. Therefore, additional knowledge regarding the use of traditional tobacco, alcohol, or other drugs among women who used e-cigarettes should be obtained and controlled for in future studies.

When defining unfavorable outcomes based on extended maternal hospital stay, we considered stays of five or more days as an unfavorable outcome, since women who have cesarean sections usually stay up to four days. Survey responses regarding the length of stay were collected in groups, one of them being “3 to 5 days.” We were unable to further divide this category and thus were forced to group extended postnatal hospital stay as any period of time greater than five days. This potentially caused an underestimation of unfavorable birth outcomes.

Participants were categorized as 1) in/did not finish high school, 2) finished high school, or 3) higher education. This delineation was intended to present the data in an objective manner, irrespective of the participant's age. We were unable to further divide this category into four groups, primarily separating those who are still in high school from those who did not finish high school. Understanding the inherent heterogeneity of these groups, future studies could consider more granular delineations for education, such as clarifying this separation or other specifications of higher education. 

A final limitation of the study was that questionnaire responses specific to e-cigarette use during pregnancy were self-reported. Participants may have provided inaccurate information leading to potential over- or underestimation of e-cigarette use. Additionally, the limitations of PRAMS were inherited in this study, primarily recall and non-response biases.

## Conclusions

In summary, our study found a positive association between e-cigarette use during pregnancy and unfavorable birth outcomes. We also found that the odds of unfavorable birth outcomes increase among women who reported having high blood pressure during pregnancy, those receiving adequate plus prenatal care, and women ages <19 years old. After adjustment, covariates, such as age, race, Hispanic ethnicity, insurance provider, maternal education, level of prenatal care, and abuse and complications during pregnancy, still had an association with unfavorable birth outcomes. There is still much that is unknown regarding the use of e-cigarettes and their long-term health effects. As e-cigarette usage continues to be pervasive in today's world, the effects of such usage and public health implications thereof present ever-growing areas for additional research.
